# Pharmacist-Driven Strategies for Hypertension Management in Los Angeles: A Community and Stakeholder Needs Assessment, 2014–2015

**DOI:** 10.5888/pcd14.160423

**Published:** 2017-07-06

**Authors:** Noel C. Barragan, Amelia R. DeFosset, Jennifer Torres, Tony Kuo

**Affiliations:** 1 Division of Chronic Disease and Injury Prevention, Los Angeles County Department of Public Health, Los Angeles, California; 2 Department of Social Welfare, Luskin School of Public Affairs, University of California, Los Angeles, Los Angeles, California; 3 Department of Epidemiology, Fielding School of Public Health, University of California, Los Angeles, Los Angeles, California; 4 Department of Family Medicine, David Geffen School of Medicine at University of California, Los Angeles, Los Angeles, California

## Abstract

In 2014, the Los Angeles County Department of Public Health received federal funding to improve the prevention and control of hypertension in the population through team-based health care delivery models, such as pharmacist-led medication therapy management. To inform this work, the department conducted a 3-part needs assessment consisting of 1) a targeted context scan of regional policies and efforts, 2) a key stakeholder survey, and 3) a public opinion internet-panel survey of Los Angeles residents. Results suggest that political will and professional readiness exists for expansion of pharmacist-led medication management strategies in Los Angeles. However, several infrastructure and economic barriers, such as a lack of sufficient payment or reimbursement mechanisms for these services, impede progress. The department is using assessment results to address barriers and shape efforts in scaling up pharmacist-led programming in Los Angeles.

## Background

Approximately 75 million Americans aged 18 years or older have hypertension, about half of whom have their hypertension under control (1). The Healthy People 2020 goal is to increase the proportion of adults with hypertension whose blood pressure is under control to 61.2% by 2020 (2). As part of its comprehensive efforts to improve hypertension management across the country, the Centers for Disease Control and Prevention (CDC) launched the 1422 State and Local Public Health Actions to Prevention Obesity, Diabetes, and Heart Disease and Stroke initiative (the 1422 initiative) (3). The initiative comprises 15 strategies, 2 of which focus on the use of team-based care models for hypertension prevention and control: 1) increase engagement of nonphysician team members in hypertension management, and 2) increase engagement of pharmacists in providing medication therapy management (MTM) for adults with high blood pressure. Pharmacist-led MTM is a distinct service or group of services that optimize therapeutic outcomes for individual patients (4). It is an example of a health care model built on team-based care principles that are effective in chronic disease management (5–7). Based on strong evidence that blood pressure control improves when a pharmacist is included in team-based care and the potential of MTM in supporting other chronic disease conditions, the 1422 initiative incorporated strategies designed to help scale up and spread these pharmacist-led interventions and related team-based care approaches in the United States (3,8). In 2014, the Los Angeles County Department of Public Health (DPH) became one of 4 large city jurisdictions funded by CDC to administer the 1422 initiative.

To inform programs to address the strategies outlined above, DPH conducted a 3-part community and stakeholder needs assessment, focusing on ways to scale up MTM and comprehensive medication management (CMM) — an advanced, evidence-based patient-centered MTM model — in both community and clinical settings. This article presents findings from the assessment and describes key needs and assets that can help steer efforts to improve hypertension prevention and control in Los Angeles and other jurisdictions.

## Community and Stakeholder Needs Assessment

Guided by the ecological approach, DPH collected information at the policy, organizational/community, and individual level (9). The needs assessment was implemented in fall 2015. It comprised 1) a context scan of existing regional policies and efforts, 2) a key stakeholder survey, and 3) a public opinion internet-panel survey of Los Angeles residents.

### Part I: Context scan of existing regional polices and efforts

To understand the political and contextual landscape in which the strategies of the 1422 initiative would be implemented, the DPH team conducted a purposeful context scan of 1) state laws on licensed pharmacists’ scope of practice and 2) public health and community-based programs aimed at promoting pharmacist-led MTM/CMM. We identified policies, programs, and initiatives as a result of conversations with subject matter experts.

#### State laws on licensed pharmacists’ scope of practice

Pharmacy practice in California made a critical shift in 2013 with the passing of Senate Bill 493 (10). The bill declared pharmacists to be “health care providers” who can bill for services, allowed pharmacists to independently initiate and administer certain medications and immunizations per state protocols, and authorized an advanced practice pharmacist (APP) board recognition program. Once fully established, the APP program will allow certified APPs to perform patient assessments, refer patients to other health care providers, and coordinate with patients’ physicians to participate in the evaluation and management of disease. At present, pharmacists in California are allowed to perform many of these tasks under collaborative practice agreements made with individual physicians and health care providers (11). However, the APP program will give them these rights without requiring collaborative practice agreements, ultimately allowing pharmacists greater flexibility in providing MTM/CMM services. Although Senate Bill 493 is an important step forward for the pharmacist community, the infrastructure and mechanisms needed to carry out such programs are mostly undeveloped; for example, efforts to develop and adopt the protocols needed to enact Senate Bill 493 continue nearly 3 years after the bill was signed into law (12).

#### Public health and community-based programs

Many agencies in California have begun to test or scale up MTM/CMM approaches. The California Department of Public Health released a report in 2015 describing some of these efforts (13). The report discussed the unique education, training, and credentialing needs of pharmacists and offered evidence of the favorable impact of CMM on patient outcomes. The report highlighted several pilot projects, including a project in the Los Angeles area that provided CMM services to high-risk patients in one of the country’s largest federally qualified health centers.

Other community-based efforts targeting low-income neighborhoods in Los Angeles have incorporated similar MTM/CMM strategies, including the LA Barbershop project in which African American men (aged 35–79 y) were screened and referred for hypertension management during their usual barbershop visits (R. Victor, A. Reid, R. Elashoff, unpublished data, 2015). Finally, MTM/CMM approaches have been promoted by nonprofit organizations such as the American Heart Association, which recently established the Western States Affiliate Blood Pressure Task Force to help states improve management of high blood pressure in vulnerable populations. One of the principal goals of the task force is to study the potential impact of MTM/CMM on health care and medical practices in health systems in in California (14).

### Part II: Key stakeholder survey

In October 2015, DPH collaborated with the University of Southern California School of Pharmacy and its partners to host the first annual pharmacist leadership symposium on opportunities to align advanced community pharmacy practice with unmet healthcare needs. The event brought together representatives from retail chain pharmacies, independent community pharmacies, academia, professional organizations, nonprofit organizations, insurance companies and other payers, and local health departments. Presentations and discussions at the symposium centered on opportunities for pharmacists to meet the chronic disease needs of communities and strategies to effectively scale up advanced pharmacy practices such as MTM/CMM in Los Angeles.

After the symposium, all 56 attendees were asked to complete a 17-item paper questionnaire developed by DPH staff; the items were informed by a literature review. The questionnaire included both closed- and open-ended questions and took approximately 10 minutes to complete (Box 1). Questions captured data on participant perspectives on priority actions needed to scale up pharmacist-led patient care activities, organizational readiness for implementing such systems or models of practice, and barriers to delivering MTM/CMM services. Collected data were managed and tallied by using Microsoft Excel software (Microsoft Corporation). This program improvement project was considered an exempt activity by the Los Angeles County DPH institutional review board.

**Box 1. Selected Questions From Key Stakeholder Survey, Administered to Attendees of the Symposium on Opportunities to Align Advanced Community Pharmacy Practice with Unmet Healthcare Needs, University of Southern California, October 2015**

Q7. Of these options, which do you consider to be the 3 most important actions to consider regarding pharmacists’ patient care services in California?

• Increase **health care provider** awareness of and receptivity to pharmacists’ patient care services

• Increase **patient **awareness of and receptivity to pharmacists’ patient care services

• Improve reimbursement procedures or options among private insurers for pharmacists’ patient care services

• Advance federal policy at Centers for Medicare & Medicaid Services to expand coverage of pharmacists’ patient care services

• Build support among health care institutions by highlighting the business case for pharmacists’ patient care services

• Standardize and increase access to training of pharmacists for advanced patient care practices

• Scale the use of collaborative practice agreements to expand pharmacists’ patient care services in the community

Q11. Please indicate the extent to which your organization has implemented the following systems or practices: [Response options: fully in place, partially in place, under development, not in place, don’t know].

Q12. If not already in place, how feasible would it be to implement or scale these systems or practices within your organization? [Response options: very feasible, somewhat feasible, somewhat not feasible, not at all feasible]

Q13. Even if not currently in place, how important are each of these systems or practices to improve patient outcomes related to medication and chronic disease management? [Response options: very important, somewhat important, somewhat not important, not important at all]

Q14. What, if any, barriers or challenges exist to implementing or scaling any of the above practices within your organization? Please explain.

[Systems or practices referenced in questions 11–14]

• Mechanisms to perform or obtain necessary assessments of a patient’s health status (eg, in-person assessments in private or semi-private settings)

• Comprehensive medication therapy reviews (MTRs) to identify, resolve, and prevent medication-related problems, including adverse drug events

• Systems to provide patients with personal medication records (PMRs) that catalog prescription and nonprescription medications, herbal products, and other dietary supplements to assist in medication therapy self-management

• Verbal education and training designed to enhance patient understanding and track progress of self-management

• Mechanisms to provide information, support services, and other resources designed to enhance patient adherence to therapeutic regimens

• Systems to monitor and evaluate the patient’s response to therapy, including safety and effectiveness

• Consulting services and interventions to address medication-related problems, including referral to a physician or other health care professional when necessary

• Systems to document care delivered and communicate essential information to the patient’s other primary care providers

• Coordination and integration of MTM [medication therapy management] services within the broader health care management services being provided to the patient

Of 56 attendees, 26 (46%) completed the survey; not all respondents answered all questions. Thirteen survey respondents reported their level of experience as at least 11 years or more, and 11 respondents self-identified as a pharmacist or as a member of pharmacy leadership in California. Respondents rated the following as top priority actions for scaling up pharmacist-led patient care services: 1) improve reimbursement procedures or options among private insurers, 2) advance federal policy at the Centers for Medicare & Medicaid Services to expand coverage of pharmacists’ services, and 3) increase health care provider awareness of and receptivity to pharmacists’ services. Professional practices and patient care system elements (eg, systems to communicate with primary care providers, patient education to support self-management, mechanisms to obtain patient health information) were consistently rated as important (Table 1). However, participants were not as optimistic about the current level of implementation in practice or the feasibility of implementing these models of practice.

**Table 1:** Responses to a Questionnaire on Implementation of Current Pharmacy Practices, Feasibility of Implementing Future Actions, and Perceived Importance to Patient Outcomes, Pharmacy Leadership Symposium, Los Angeles County, 2015^a^

Pharmacy Practice	Answered “Fully” or “Partially” Implemented (%)	Answered “Very” or “Somewhat” Feasible to Implement (%)	Answered “Very” or “Somewhat” Important in Improving Patient Outcomes (%)
Mechanisms to perform or obtain assessments of patient's health status (eg, in-person assessments in private or semi-private settings)	10 of 21 (47.6)	12 of 15 (80.0)	19 of 19 (100.0)
Comprehensive medication therapy reviews to identify, resolve, and prevent medication-related problems, including adverse drug events	13 of 21 (61.9)	13 of 16 (81.3)	19 of 19 (100.0)
Systems to provide patients with personal medication records that catalog prescription and nonprescription medications, herbal products, and other dietary supplements to assist in medication therapy self-management	11 of 21 (52.4)	13 of 16 (81.3)	19 of 19 (100.0)
Verbal education and training designed to enhance patient understanding and track progress in self-management	10 of 20 (50.0)	15 of 17 (88.2)	19 of 19 (100.0)
Mechanisms to provide information, support services, and other resources designed to enhance patient adherence to therapeutic regimens	11 of 21 (52.4)	14 of 16 (87.5)	19 of 19 (100.0)
Systems to monitor and evaluate the patient’s response to therapy, including safety and effectiveness	11 of 20 (55.0)	16 of 16 (100.0)	19 of 19 (100.0)
Consulting services and interventions to address medication-related problems, including referral to a physician or other health care professional when necessary	13 of 21 (61.9)	13 of 15 (86.7)	19 of 19 (100.0)
Systems to document care delivered and communicate essential information to the patient’s primary care providers	12 of 21 (57.1)	12 of 15 (80.0)	19 of 19 (100.0)
Coordination and integration of medication therapy management services within the broader health care management services being provided to the patient	12 of 21 (57.1)	14 of 16 (87.5)	19 of 19 (100.0)

a Twenty-six of 56 symposium attendees completed the 17-item survey. Not all respondents answered all questions; denominators indicate the number of participants who answered the question.

Respondents identified many barriers associated with the scale-up and spread of pharmacist-led patient care services: 7 respondents indicated reimbursement and funding challenges; 6 respondents indicated health care provider challenges (ie, need for increased physician/provider awareness, support, and coordination); and 5 respondents indicated limited electronic record capabilities (eg, need for electronic medical record systems that readily allow for pharmacist–provider communications).

### Part III: Public opinion internet-panel survey

#### Survey methods

In December 2015, DPH commissioned Global Strategy Group to conduct a clinical services internet-panel survey of adult residents of Los Angeles. DPH developed the survey questions with support from Global Strategy Group, drawing from nationally validated surveys and internally developed instruments. Data were collected during 2 weeks and included data on demographics, health behaviors and attitudes, opinions of health care providers (eg, pharmacists), and personal health status. The survey was administered in English and Spanish to adults (aged ≥18 y) who resided in Los Angeles. Participants were recruited from existing participant panels established by reputable panel providers via email, social media, and mobile telephone applications. Incentives were provided after survey completion based on a structured incentive schedule established by the panel provider; rewards were determined based on the length of the survey and could be redeemed for miles, gift cards, or other items. All collected data were weighted to account for differential sampling rates, differential nonresponse, and other variables (marital status, education, income, and other demographic distributions of Los Angeles County). Data for demographic weights were based on the 2013 American Community Survey (15) and the 2011 Los Angeles County Health Survey (16).

For the pharmacist-led MTM component of the survey, participants were asked 2 questions and provided with the following definition of pharmacist-led MTM: “Medication therapy management (MTM) is a medical service provided to patients by pharmacists to optimize drug and improve therapeutic outcomes. MTM includes a broad range of professional activities, including but not limited to performing patient assessment and/or a comprehensive medication review, formulating a medication treatment plan, monitoring efficacy and safety of medication therapy, enhancing medication adherence through patient empowerment and education, and documenting and communicating MTM services to prescribers in order to maintain comprehensive patient care.” The 2 questions were 1) “To your knowledge, do you have access to MTM [medication therapy management] at the place where you usually go or last went for health care?” and 2) “If MTM were available where you currently go for health care, how interested would you be in receiving this service when you need to take medicine? If you have used MTM please check that box.” All study materials were reviewed and approved by the DPH institutional review board before field implementation.

#### Data analysis and survey results

We generated descriptive statistics to describe participant demographics and understand the response profiles of those who were aware of having access to MTM services and would be interested in receiving MTM services. To further explore participant interest in receiving MTM services (dependent variable), we performed binary logistic regression. Model covariates, which were entered at the same time in the final model, included demographic characteristics (ie, age, sex, race/ethnicity, education, relationship status, insurance status), health indicators (ie, number of chronic conditions ever diagnosed, self-reported health status), knowledge of having access to MTM, and level of comfort discussing health issues with pharmacists. All data analyses were conducted by using StataSE version 14.0 (StataCorp LP).

Of 33,766 people initially invited to participate in the internet-panel survey, 1,751 clicked on the survey link. Among those who clicked on the link, 737 people were excluded because 1) they did not meet survey criteria or quotas established by Global Strategy Group to ensure accurate representation of the Los Angeles population (n = 460), 2) they did not complete the survey (n = 175), or 3) they were invalidated because of speeding (when respondents answer questions so quickly that they probably are not thoughtfully answering the questions) or straight lining (when respondents choose the same response for every question and are probably not thoughtfully answering the questions) (n = 102). Our analytic sample consisted of 1,014 participants. Approximately 10% of the data were missing; only those with complete data (n = 968) were included in the model analysis.

Most participants were aged 25 to 64 (71.2%), were Hispanic (42.8%) or white (30.4%), and reported being in excellent or very good health (55.9%) (Table 2). Approximately one-third (34.8%) reported having at least 2 chronic conditions. Approximately 9% reported having access to MTM services where they usually go for care, and 41.3% expressed interest in using or having used MTM services, regardless of what was currently available. Among participants who expressed interest in using MTM, 51.2% were women, 54.0% reported excellent to very good health, and 85.5% said they were generally comfortable speaking to a pharmacist.

**Table 2:** Participant Demographics, Access to MTM, and Interest in Receiving MTM Services: Results of a Los Angeles County Internet-Panel Survey, 2015

Characteristics	No. (Weighted Proportion^a^) (n = 1,014)
**Age, y**	
18–24	107 (14.2)
25–44	407 (39.2)
45–64	334 (32.0)
≥65	166 (14.6)
**Sex**	
Male	454 (48.7)
Female	560 (51.3)
**Race/ethnicity**	
White	417 (30.4)
Hispanic	317 (42.8)
African American	69 (8.4)
Asian	184 (16.2)
Other	27 (2.3)
**Marital status**	
Married	429 (45.9)
Not married, but living with partner	91 (7.2)
Single	359 (35.3)
Divorced/separated/widowed	128 (11.0)
Prefer not to say	7 (0.6)
**Insurance status**	
Employer provided	480 (45.4)
Self-purchased	127 (11.3)
Medicare	182 (17.8)
Medicaid	139 (15.5)
Military	7 (0.5)
Other/don’t know	79 (9.6)
**Education**	
High school diploma or less	183 (30.5)
Some college or technical school	227 (25.1)
Associate’s degree	75 (8.5)
Bachelor’s degree	324 (21.9)
Graduate degree	198 (13.3)
Prefer not to answer	7 (0.7)
**Self-reported health status**	
Excellent/very good	587 (55.9)
Good/fair	413 (42.4)
Poor	14 (1.6)
**No. of chronic conditions ever diagnosed**	
0 or 1	649 (65.2)
2 or 3	247 (23.0)
≥4	118 (11.8)
**Comfort speaking to pharmacist**	
Extremely comfortable	119 (13.6)
Very comfortable	259 (24.2)
Somewhat comfortable	404 (38.6)
Not very comfortable	146 (14.6)
Not at all comfortable	86 (9.1)
**Do you have access to MTM at the place where you usually go or last went for care?^b^**	
Yes	76 (9.1)
No	267 (25.3)
Don’t know/not sure	671 (65.6)
**If MTM were available, how interested in receiving the service when you need to take medication?^b^**	
Very interested	77 (8.1)
Somewhat interested	317 (31.0)
Not very interested	308 (28.5)
Not interested at all	292 (30.2)
Have used MTM	20 (2.2)

Abbreviation: MTM, medication therapy management.

a All collected data were weighted to account for differential sampling rates, differential nonresponse, and other variables (marital status, education, income, and other demographic distributions of Los Angeles County). Data for demographic weights were based on the 2013 American Community Survey (15) and the 2011 Los Angeles County Health Survey (16).

b Survey participants were provided with the following definition of pharmacist-led MTM: “Medication therapy management (MTM) is a medical service provided to patients by pharmacists to optimize drug and improve therapeutic outcomes. MTM includes a broad range of professional activities, including but not limited to performing patient assessment and/or a comprehensive medication review, formulating a medication treatment plan, monitoring efficacy and safety of medication therapy, enhancing medication adherence through patient empowerment and education, and documenting and communicating MTM services to prescribers to maintain comprehensive patient care.”

The binary logistic model indicated that older age (≥65 y) predicted interest in MTM (*P* = .02). The model also indicated that those who were aware of having access to MTM services were less likely than those who had no knowledge to express interest in receiving MTM services (*P* < .001). Additionally, compared with those who felt comfortable talking to pharmacists about their health, those who were not comfortable speaking with their pharmacists were more likely to be interested in receiving MTM services (*P* < .001). Although somewhat unexpected, these results align with research on the challenges of developing client interest in MTM services and the complexities of patient decision making (17,18). Factors that inform patients’ decision making are complex, and the process is often influenced not only by the perceived value of an intervention but also by the level of perceived harm from their condition (19,20). Although more research is needed on patients’ level of comfort in talking to pharmacists, patients who are not comfortable talking to an individual pharmacist may perceive the team-oriented MTM as a desirable alternative.

## Discussion

Our needs assessment suggests challenges and opportunities for scaling up pharmacist-led MTM/CMM interventions. First, legislation (ie, Senate Bill 493) supports advancing MTM/CMM practice. However, infrastructure for expanding the practice is lacking. Second, many in the pharmacist community are ready to take action to scale up and spread MTM/CMM, but the lack of mechanisms for reimbursement of more advanced pharmacist practices is a key barrier to expansion. Third, although many people report feeling comfortable discussing health issues with their pharmacist, this comfort level does not necessarily translate into interest in MTM services. Our assessment also led to the creation of a synthesized list of needs and assets (Box 2). This information could be useful for informing the scale-up and spread of MTM/CMM programming in Los Angeles and elsewhere in the United States.

**Box 2. Synthesis of Needs and Assets Associated With the Scale-Up and Spread of Medication Therapy Management and Comprehensive Medication Management Programming in Los Angeles^a^**

**Needs**

Interoperable electronic medical record systems that facilitate pharmacist and provider communication (ie, capability to share information across different software platforms).

Clinic workflows that facilitate integration of pharmacists into primary care settings.

Payment and reimbursement reform for pharmacists, particularly in the community setting.

Increased health care provider awareness of and receptivity to pharmacists’ patient care services (eg, calm fears among health care providers of losing patients to other providers).

Increased leadership or champions at all levels of practice advocating for integration of pharmacists within team-based care models.

Increased patient awareness and receptivity to the broadened scope of work of pharmacists in the health care team.

**Assets**

Pharmacists represent a highly skilled workforce that is currently underutilized and is ready and willing to expand their contributions to the health care team.

Federal and state support for integrating pharmacists into health care teams (ie, Patient Protection and Affordable Care Act, Senate Bill 493 in California).

Emerging evidence of the positive impact of increasing the role of pharmacists on the health care team and resultant best practices from pilot projects in diverse populations.

Overall public familiarity with pharmacists and comfort working with them.

With a growing demand for primary care services, there is increased opportunity to demonstrate the potential value in incorporating pharmacists more broadly into team care models.

Designation of pharmacists as health care providers in California, allowing for increased opportunities to establish reimbursement mechanisms for an expanded scope of work.

^a^ Data sources: Butler et al (13), Los Angeles County Department of Public Health.

Although this community and stakeholder needs assessment provides insights into readiness to scale up MTM/CMM strategies in Los Angeles, it has limitations. First, the context scan of MTM/CMM efforts in Los Angeles and across California was not exhaustive; it was purposefully focused on legislative and programmatic strategies. Second, results from the leadership symposium survey offered only a snapshot of pharmacist and public health leadership opinions and did not capture data on the viewpoints of other health care professionals (eg, physicians, nurses). Other viewpoints may be important, because key processes in the health care system are not under the purview of pharmacy or public health communities. Third, the sample size was small and represented a group of providers who self-selected to attend a meeting promoting the use of MTM/CMM, potentially biasing the results of the survey. Finally, the internet-panel survey posed challenges to precise interpretation of public support for MTM services. Survey limitations include the following: 1) the recruitment mechanism used by internet-panel surveys lends itself to a high nonresponse rate, which could have limited the survey’s validity; 2) although most Los Angeles residents speak mostly English or Spanish at home, participant views may differ and not reflect the views of other populations; 3) because the survey was internet based, people who have a limited understanding of MTM/CMM programming and its definition may have been underrepresented; 4) questions assessing interest in MTM did not provide qualifying information such as cost or scope of MTM; and 5) the quantitative nature of the survey did not allow for exploration of participant reasoning for their interest or lack thereof.

Public health and other health professions can capitalize on the opportunities identified in our needs assessment to better coordinate care for hypertension management in the community. Lessons learned from the effort in Los Angeles can inform other jurisdictions interested in strengthening its infrastructure for MTM/CMM programs.
